# Long-Term Effects of Pediatric Acute Lymphoblastic Leukemia Chemotherapy: Can Recent Findings Inform Old Strategies?

**DOI:** 10.3389/fonc.2021.710163

**Published:** 2021-10-15

**Authors:** Zeina N. Al-Mahayri, Mohammad M. AlAhmad, Bassam R. Ali

**Affiliations:** ^1^ Department of Genetics and Genomics, College of Medicine and Health Sciences, United Arab Emirates University, Al-Ain, United Arab Emirates; ^2^ Department of Clinical Pharmacy, College of Pharmacy, Al-Ain University, Al-Ain, United Arab Emirates; ^3^ Zayed Center for Health Sciences, United Arab Emirates University, Al-Ain, United Arab Emirates

**Keywords:** pediatric acute lymphoblastic leukemia, long-term chemotherapy adverse events, secondary tumors, cardiotoxicity, neurotoxicity, cancer survivors

## Abstract

During the last few decades, pediatric acute lymphoblastic leukemia (ALL) cure rates have improved significantly with rates exceeding 90%. Parallel to this remarkable improvement, there has been mounting interest in the long-term health of the survivors. Consequently, modified treatment protocols have been developed and resulted in the reduction of many adverse long-term consequences. Nevertheless, these are still substantial concerns that warrant further mitigation efforts. In the current review, pediatric-ALL survivors’ late adverse events, including secondary malignant neoplasms (SMNs), cardiac toxicity, neurotoxicity, bone toxicity, hepatic dysfunction, visual changes, obesity, impact on fertility, and neurocognitive effects have been evaluated. Throughout this review, we attempted to answer a fundamental question: can the recent molecular findings mitigate pediatric-ALL chemotherapy’s long-term sequelae on adult survivors? For SMNs, few genetic predisposition factors have been identified including *TP53* and *POT1* variants. Other treatment-related risk factors have been identified such as anthracyclines’ possible association with breast cancer in female survivors. Cardiotoxicity is another significant and common adverse event with some germline variants been found, albeit with conflicting evidence, to increase the risk of cardiac toxicity. For peripheral neurotoxicity, vincristine is the primary neurotoxic agent in ALL regimens. Some germline genetic variants were found to be associated with the vincristine neurotoxic effect’s vulnerability. However, these were mainly detected with acute neuropathy. Moreover, the high steroid doses and prolonged use increase bone toxicity and obesity risk with some pharmacogenetic biomarkers were associated with increased steroid sensitivity. Therefore, the role of these biomarkers in tailoring steroid choice and dose is a promising research area. Future directions in pediatric ALL treatment should consider the various opportunities provided by genomic medicine. Understanding the molecular bases underlying toxicities will classify patients into risk groups and implement a closer follow-up to those at higher risk. Pharmacogenetic-guided dosing and selecting between alternative agents have proven their efficacy in the short-term management of childhood ALL. It is the right time to think about a similar approach for the life-long consequences on survivors.

## 1 Introduction

Pediatric acute lymphoblastic leukemia (ALL) is the most common type of cancer in children. During the last decades, pediatric ALL cure rates have improved to exceed 90% of patients, with parallel progress in event-free and 5-years survival rates (1). Current treatment modalities applied since the 1990s and beyond stratified ALL patients into risk groups and tailored treatment accordingly. This strategy succeeded in improving outcomes significantly and reduced adverse treatment sequelae. Older treatment regimens, used during the seventies and eighties of the last century, involved cranial radiotherapy (CRT) for all patients to prevent central nervous system relapse. Newer regimens replaced CRT with intrathecal chemotherapeutic regimens and high doses of intravenous chemotherapy. Likewise, chemotherapy’s intensity has changed with time by decreasing anthracycline concentrations but increasing asparaginase, dexamethasone, and high dose methotrexate (2).

Parallel to this drastic improvement in pediatric ALL and other types of childhood cancer outcomes, there has been a growing interest in the survivors’ health and well-being. It is estimated that in the United States alone there are currently almost 500,000 adults who survived childhood cancer (i.e., one childhood cancer survivor (CCS) for every 750 individuals). By the age of 40, most of these survivors are anticipated to develop at least one chronic condition. These figures stimulated re-examination of treatment regimens’ long-term consequences by applying longitudinal studies on survivors (3).

One of the most extensive longitudinal studies is the Childhood Cancer Survivor Study (CCSS) (https://ccss.stjude.org/). CCSS is a retrospective study carried by thirty-one USA research centers to investigate the long-term effects of treatment among cancer survivors diagnosed between 1970 and 1999. Siblings of a random group of the recruited survivors were also included as a comparison cohort (4). Although the CCSS did not conclude its final results yet, several groups exploited its data to analyze cancer treatment’s long-term effects in children. Gibson and colleagues found a significant decrease of the 20-years cumulative incidence of grade 3-5 chronic conditions between cancer survivors treated in the 1970s and those treated in the 1990s (incidence rates 33.2% and 27.5%, respectively). Nevertheless, this incidence is still significantly higher than the survivors’ aged-matched siblings (incidence rates 4.6%) (5).

A more recent analysis from the same dataset reported a significant decrease in mortality rates among contemporary pediatric ALL survivors than the ALL 1970s survivors. Moreover, Dixon and colleagues reported no significant difference in pediatrics mortality rates with standard-risk ALL (SR-ALL) treated in the 1990s compared to the general population. Still, an increase in chronic health conditions was significant for patients treated in the 1990s with the high-risk ALL (HR-ALL) protocols. Similarly, higher rates of specific chronic conditions were reported for ALL patients who received bone marrow transplants and those who suffered from relapse (BMT/R-ALL). The pronounced chronic morbidities with increased incidence rates included joint replacement, diabetes, and heart failure. The authors concluded that the long-term outcomes of the risk-stratified therapy followed in the 90s protocols are encouraging for SR-ALL survivors. However, noticeably increased specific chronic adverse effects were reported for HR-ALL and BMT/R-ALL patients and warrant further mitigation efforts (2).

In response to recognizing the high incidence of chronic mortalities among cancer survivors, the Children’s Oncology Group (COG) issued long-term follow-up guidelines for practitioners, known as COG LTFU guidelines (http://www.survivorshipguidelines.org/) according to the type of cancer therapy the patient received during childhood (6). However, the literature supporting these recommendations for pediatric ALL survivors is heterogeneous, and the recent molecular findings are not well-integrated.

The current review will describe the broad spectrum of long-term adverse events, as listed in the COG LTFU, the evidence supporting associations of these events with ALL treatment, their etiology, possible risk factors, and the latest findings of predictive biomarkers. Throughout the following sections, we try to answer a fundamental question: can the most recent molecular and pharmacogenetic results be used to mitigate pediatric-ALL chemotherapy’s long-term sequelae on adult survivors?

## 2 Long-term Morbidities Following Pediatric-ALL Chemotherapy Treatment

**Figure 1:**
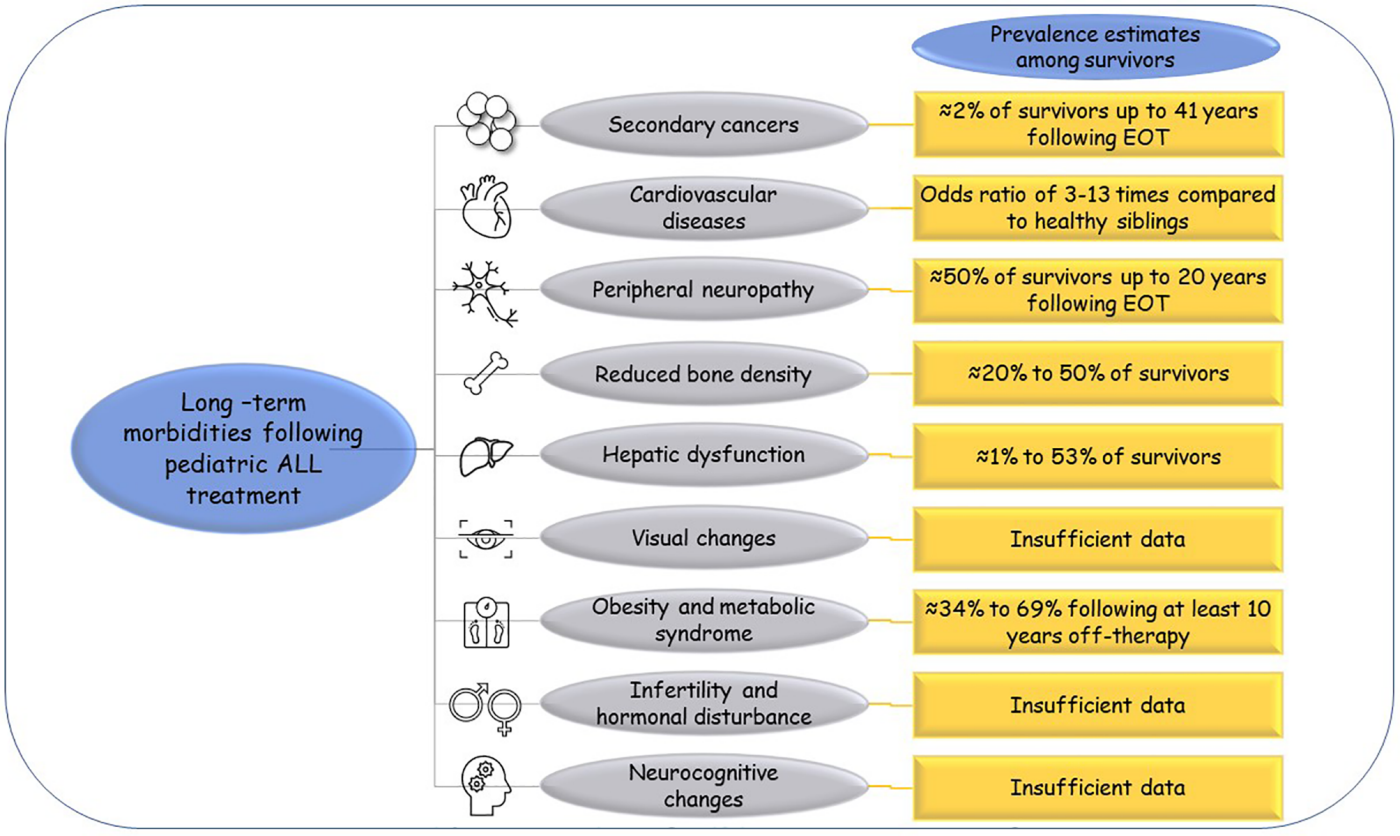
Long-term morbidities following pediatric ALL treatment. The commonly reported long-term morbidities for pediatric ALL survivors and their estimated prevalence as reported from large studies like the CCSS. EOT, end of therapy.

Reports from the CCSS indicated that ALL survivors have higher risks of early mortalities at 25 years from diagnosis than their sibling controls. Besides relapse, multiple morbidities have been attributed to the survivors’ decreased quality of life and as death causes. The reported long-term morbidities include secondary malignancies, cardiac, neurological, and endocrine disorders (7). **Figure 1** illustrates these morbidities for ALL survivors and their estimated prevalence inferred from multiple reports. The order of the following paragraphs does not indicate how the listed events are prevalent among ALL survivors.

### 2.1 Secondary Cancers

Cancer survivors are more likely to develop second malignant neoplasms (SMNs) than the general population by four to six folds (8). SMNs include neoplasms occurring at any site with a morphology other than that of the primary cancer. In data collected from 3464 cancer survivors from the UK, 2% of those were diagnosed with at least one SMN (9). SMNs usually occur with more aggressive clinical manifestations and are harder to treat with worse outcomes than their primary counterparts (10). The highest risk of a second cancer is observed within the first five years following the first cancer diagnosis; however, the risk remains elevated over 20 years (11).

In a recent analysis of 24,403 pediatric leukemia cases, 1.81% of the survivors developed a secondary malignancy within a time frame of 0 to 41 years (median follow-up time = 13 years). The most common secondary malignancy for leukemia survivors was secondary leukemia (23.9%), followed by thyroid cancer (18.33%), sarcoma (15.14%), astrocytoma (10.36%), lymphoma (9.56%), salivary gland carcinoma (7.17%), melanoma (4.38%), breast cancer (3.98%), followed by other less common types (12). Older estimates reported quite different prevalence of SMN types, with acute myelogenous leukemia (AML), myelodysplastic syndrome (MDS), and brain tumors as the most common ones in pediatric ALL survivors (13). Regardless of the type of SMNs, these events, although rare, are devastating complications for survivors and their families (12).

Radiotherapy is a well-established risk factor for SMNs induction, but chemotherapeutic agents were later found to be independent risk factors (11). For example, anthracyclines exert their cell-killing activity through targeting topoisomerase II (Top2). There are two isoforms of Top2, Top2α, and Top2β, which have different functions in human cells. While anthracyclines interfere with both isoforms, the effect on Top2α is considered the primary basis of their activity as this isoform is central to cell proliferation and replication. On the other hand, the impact on Top2β has been associated with long-term adverse events, including secondary tumors and cardiotoxicity. Currently, it is believed that the poisoning effects of anthracyclines on Top2β can induce therapy-related chromosomal translocations, leading to secondary cancers (14). One of the best-established associations between chemotherapy exposure and SMN is between anthracyclines and therapy-related AML and MDS (15).

**Figure 2:**
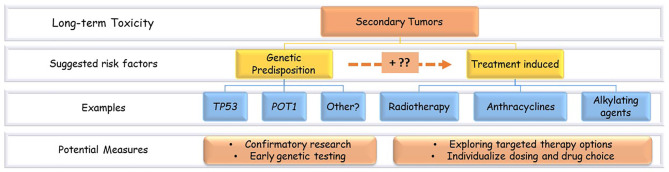
Secondary tumors risk factors and potential measures. Examples of the suggested risk factors of secondary tumors in ALL survivors. There is a probable interaction between genetic and treatment-related factors.

Besides the treatment-related risk factors, the increased risk of second tumors in childhood cancer survivors might be related to the constitutional genetic predisposition to cancers (**Figure 2**). For example, germline mutations in the tumor suppressor p53 gene (*TP53*) that characterize Li Fraumeni syndrome (LFS) are known to increase the risk of SMNs (10). Nevertheless, harboring such predisposing variants can boost the tissue sensitivity to the treatment’s toxic effects (11).

Regarding genetic predisposition mutations, ALL survivors should be considered with more concern. ALL is one of the LFS-associated cancers, and almost 50% of pediatric-ALL patients who are hypodiploid at diagnosis harbor germline *TP53* mutations (16). Besides poor prognosis and low survival rates, *TP53* variants in ALL were associated with SMN. These findings suggest that analyzing germline *TP53* mutations during childhood cancer diagnosis can identify patients at higher risk for developing SMNs later in life. Patients found to have germline predisposition can benefit from modified therapy to neutralize their SMN risk. In their study that comprehensively characterized *TP53* mutations in more than 3000 pediatric ALL patients, Qian and colleagues suggested the use of non-genotoxic agents, for example, immunotherapies, for patients carrying these mutations as a measure to reduce SMN induction (17). However, developing such new approaches is undoubtedly a complicated process that requires strong evidence. Meanwhile, patients identified with *TP53* mutations can be offered a closer SMN surveillance following cure. The current recommendations from the COG LTFU and the National Comprehensive Cancer Network (NCCN) guidelines focus on SMN surveillance in patients treated with high radiation doses (10). Germline *TP53* mutations are listed as risk factors for SMNs; however, testing for these mutations is still not a routine practice (10).

Thyroid secondary neoplasm is one of the common SMNs in pediatric leukemia survivors. Its risk increases in females, the younger incidence age of primary cancer, radiation, and alkylating chemotherapeutics use (18). Nevertheless, genetic factors are investigated as potential predisposing factors for thyroid SMN. Richard and coworkers found an intronic variant in *POT1* (protection of telomeres one gene) with a statistically significant association with thyroid SMN in the CCSS participants. This gene contributes to the “shelterin complex” protecting DNA telomeres ends. The germline POT1 intronic variant can disturb the latter complex, resulting in a longer and more fragile and unstable germline DNA, increasing cancer risk. However, due to data limitations, whether this genetic effect was enhanced by radiotherapy or chemotherapy is not determined yet. Confirmatory studies might be applied when a subset of cancer survivors larger than the CCSS emerges (18). If such an interaction is confirmed, *POT1* variants can be added to the list of candidate genetic biomarkers for SMNs, and interventional studies can examine the feasibility of genetic-guided treatment.

On the other hand, the effect of treatment components as SMNs inducers should be reconsidered. Female childhood cancer survivors are at a four-fold increased risk of developing breast cancer than the general population of the same age (19). Chest radiotherapy during childhood cancer treatment has been regularly ascribed to this increased risk. Henderson and colleagues showed that by the age of 45, female leukemia survivors without any history of radiotherapy have a 6.3% cumulative incidence of breast cancer. In the same study, which revised data of 3,867 female cancer survivors, from which 1,411 were ALL survivors, high doses of anthracyclines (> 250 mg/m^2^) and alkylating agents (cyclophosphamide) increased breast cancer risk to almost 10-folds. The median age at breast cancer diagnosis in this group of survivors was 38 years, which is much younger than the median age of breast cancer diagnosis in the general population (19). In another study that included more than 6000 pediatric cancer survivors, from which 2092 were leukemia survivors, doxorubicin was associated with an increased breast SMN risk in a dose-dependent manner (20).

Collectively, these findings raise awareness of the genetic component in SMNs development. Future studies should confirm the suggested biomarkers, like *TP53* and *POT1*, and investigate new ones. If such associations were proved, genetic biomarkers’ early testing would warrant closer surveillance for patients at risk. The association between specific chemotherapy agents and the development of some kinds of SMNs, like the suggested correlation between anthracyclines exposure and breast cancer, warrant closer examination and confirmatory studies that elucidate molecular and genetic mechanisms of this association followed by studies on interventional approaches. **Figure 2** illustrates the proposed risk factors of SMNs in pediatric ALL survivors and the potential prevention measures.

### 2.2 Cardiotoxicity

Cardiovascular diseases (CVD), including heart failure, arrhythmias, coronary artery diseases, and other cardiac morbidities, are the foremost concern of cancer survivors. It is estimated that one of each eight cancer survivors treated with radiotherapy or anthracyclines will develop a life-threatening CVD thirty years after treatment. Moreover, death due to CVD occurs seven more times in childhood cancer survivors compared to the general population (21).

For pediatric ALL patients, anthracyclines are the primary cardiotoxic cause. The cardiotoxicity risk increases significantly when anthracyclines’ accumulative dose exceeds 250 mg/m^2^ (21). Since the introduction of current treatment regimens, a higher percentage of patients are exposed to anthracyclines; however, most of these are treated with doses less than 250 mg/m^2^ (22). Nevertheless, patients exposed to doses lower than 250 mg/m^2^ are still vulnerable to an increased cardiotoxicity risk compared to those non-exposed with an odds ratio of 3.9 (23). The CCSS data showed that patients treated with HR-ALL regimens have a three-fold increase in heart failure than their siblings. Moreover, the increase in heart failure rates reaches 13 folds more than healthy siblings for patients treated with the BMT/R-ALL protocols (2).

Traditional cardiac risk factors, like obesity, smoking, and hyperlipidemia contribute significantly to the cardiac risk in cancer survivors (22). Other risk factors related to oncology treatment include age at diagnosis, anthracycline dose, and chest radiation. Using these variables in a prediction cohort of more than 13,000 pediatric cancer survivors and a validation cohort of more than 3,000 survivors, Chaw and colleagues built a heart failure prediction model. In this model, patients were successfully classified into low, moderate, and high-risk groups with regard to heart failure by the age of 40 with incidence rates of 0.5%, 2.4%, and 11.7%, respectively (24). Nevertheless, interindividual differences in heart failure occurrence and severity were not fully explained by the previous factors, which suggested a genomic compartment (21).

A considerable body of research investigated anthracycline-induced cardiotoxicity molecular pathogenesis; however, most applied studies utilized animal models at doses higher than those used in the clinic, limiting their findings. Moreover, the mechanisms of chronic cardiotoxicity encountered in childhood cancer survivors are probably distinct from acute and adulthood cardiac toxicity (21). Still, understanding the molecular pathogenesis of cardiac toxicity is indispensable for prioritizing genomic targets. Superoxide species accumulation leading to free radicals’ stress is the key mechanism of anthracycline-induced cardiotoxicity (25). The increased oxidative stress occurs due to different processes involving the iron regulatory protein, mitochondrial function, impaired adenosis triphosphate (ATP) levels, calcium degeneration, and other complex and intersecting pathways (26).

Moreover, the renin-angiotensin-aldosterone system (RAAS) activation plays a vital role in anthracycline-induced cardiac events. RASS is a precisely controlled system that maintains ions and fluid balance and controls blood pressure. RASS has a role in the cellular stress response at the cardiomyocytes contributing to anthracycline-induced pathological heart remodeling and chronic cardiac toxicity (25).

Building on its role in anthracycline-induced cardiotoxicity, Blanco and colleagues explored the effect of a gene interacting with oxidative stress, *NQO1* (NAD(P)H Quinone Dehydrogenase 1). This gene encodes an oxidoreductase that protects against intracellular oxidative stress. Another gene targeted in the same study was *CRB3* (Crumbs Cell Polarity Complex Component 3) which catalyzes the reduction of anthracycline side chain during its cardiotoxic alcohol metabolite formation. The findings indicated a possible role of a *CRB3* variant on the risk of anthracycline-induced cardiotoxicity in survivors of childhood cancer, without a similar association for the tested *NQO1* variant (23).

Indeed, one of the common approaches in pharmacogenomic (PGx) studies (i.e., studies carried to investigate genetic compartments in pharmacological responses) is to explore genes affecting the biotransformation of the drug(s) of interest. This approach hypothesizes that differences in active to inactive drug ratio might explain differences in drug responses and toxicities. Under this assumption, Visscher and coworkers explored variations in 220 genes involved in anthracyclines’ biotransformation. This work identified a highly significant association between a variant in a transporter gene, *SLC28A3* (Solute Carrier Family 28 Member 3): rs7853758, and anthracycline-induced cardiotoxicity in a group of cancer children followed for a median of 8.6 years. Unfortunately, the previously identified association between *CRB3* variants and cardiotoxicity failed to replicate in this group (27). A *UGT1A6* (UDP Glucuronosyltransferase Family 1 Member A6) allele, known as UGT1A6*4, has been prioritized in the same group (27), then successfully replicated in a succeeding study within an independent cohort. The second study included an independent cohort of 218 patients and examined 23 variants in different anthracycline biotransformation pathways. Again, the same variants in *SLC28A3* showed a significant association with cardiotoxicity (28).

Similarly, Semsei and colleagues hypothesized that the efflux transporter gene, *ABCC1* (ATP Binding Cassette Subfamily C Member 1), might contribute to differential anthracycline concentration at the heart tissue, affecting the drug’s cardiotoxicity. In their study, 235 pediatric ALL patients were followed for a median of 6.3 years. *ABCC1* rs3743527 and rs246221 were associated with lower left ventricular ejection fraction shortening after anthracycline treatment, contributing to cardiotoxicity development (29).

Other groups opted to explore genes involved in cardiovascular diseases occurring in the general population as plausible biomarkers for anthracycline-induced cardiac morbidities. Wang and colleagues utilized a cardiovascular array of 34,912 SNPs in 2,100 genes known to harbor heart diseases biomarkers. The study participants included 93 cases (i.e., with an anthracycline-induced cardiac disease), 194 controls, and an independent 73 patients as a validation set, all childhood cancer survivors. The homozygous carriers of a variant allele in hyaluronan synthase 3, *HAS3*, rs223228, were at an increased cardiotoxicity risk by 8.9 folds compared to wild type homozygous carriers. The authors suggested that the *HAS3* variant exerted its effect by interrupting the extent of cardiac remodeling and repair following anthracycline-induced injury (30).

In the same context, Garcia-Pavia and coworkers utilized a cardio sequencing panel in a next-generation sequencing platform, focusing on nine genes previously identified as cardiomyopathy risk genes. The study encompassed multiple cohorts of cancer patients treated with anthracyclines, of which 41 were survivors of childhood AML. The results show a significant association between cardiotoxicity and variants in *TTN* (Titin) encoding titin, which has a role in controlling stress in cardiomyocytes (31). Despite this study findings may not apply to pediatric ALL survivors; it supported earlier evidence about the contribution of cardiac morbidities’ genes to the development of anthracycline-induced cardiotoxicity, which suggests further investigation in these sets of genes.

Likewise, Hildebrandt and coworkers hypothesized that hypertension susceptibility loci might serve as chronic anthracycline-induced cardiotoxicity biomarkers. They investigated twelve previously identified hypertension loci in a cohort of 108 childhood cancer survivors. Variants in *PLCE1* (Phospholipase C Epsilon 1) and *ATP2B1* (ATPase Plasma Membrane Ca2+ Transporting 1) were found to have a protective effect from anthracycline-induced cardiotoxicity. *PLCE1* encodes a protein that reduces reactive oxygen species generation, while *ATP2B1* encodes a calcium pump that maintains calcium levels during heart relaxation (32).

Krajinovic and coworkers carried another targeted-gene design study on 251 Caucasian pediatric ALL survivors followed for more than five years (mean time since diagnosis = 8.4 years). The targeted polymorphisms were in transporter genes from the ABC family members, active in reactive oxygen and nitrogen species like NQO1, *NOS3* (Nitric Oxide Synthase 3), DNA repair proteins, and detoxifying enzymes. Associations revealed variants in *ABCC5* and *NOS3* with a modulating effect on anthracycline-induced cardiotoxicity. Both variants were suggested as possible biomarkers that need further confirmation (33).

On the other hand, multiple groups utilized the genome-wide association studies (GWAS) agonistic design to explore genetic biomarkers for anthracycline-induced cardiotoxicity rather than the targeted gene design. However, most of these studies were restricted by their small size and limited power. One of the earliest GWASs applied on 280 cancer survivors treated with an anthracycline identified a novel genetic biomarker in *RARG* (retinoic acid receptor). In the same study, functional assays suggested that patients with the *RARG* variant allele have higher basal levels of the topoisomerase, TOP2 β, in cardiomyocytes, which confers them to be more vulnerable to anthracycline cardiotoxicity (34).

Another GWAS suggested a variant in *CELF4* to have an enhancing cardiotoxic effect of anthracyclines. *CELF4* encodes for a splicing regulator that controls tissue-specific splicing events. One of the latter regulators’ common targets is the cardiac troponin T gene, *TNNT2*, which is a biochemical biomarker of myocardial injury (35). Other studies pointed out that the increase in troponin T levels in the first 90 days of doxorubicin treatment in pediatric ALL patients was associated with echocardiographic changes four years later, despite the absence of such changes at the beginning of treatment. Cardiomyocytes have limited regeneration capacity, which reflects accumulative damage with cumulative anthracycline doses. When the damage exceeds the repair mechanisms, it results in chronic heart disease progression. Accordingly, troponin T and other myocardial damage biomarkers monitoring could facilitate the early identification of vulnerable patients. Such approaches warrant deeper investigation as a reasonable predictive measure (36).

A third GWAS was applied to 93 children treated with anthracyclines for different tumors, including ALL. None of the 246,060 examined SNPs reached genome-wide statistical significance; however, a succeeding gene-based analysis identified variants in *GPR35*, G Protein-Coupled Receptor 35, as the gene most significantly associated with cardiotoxicity. This gene encodes a G-coupled receptor proposed to function in heart physiology and pathology and act as a biomarker of heart failure (37). The same group utilized an identical array to investigate chronic anthracycline-induced cardiotoxicity biomarkers in a group of breast cancer patients with a validation cohort of pediatric cancer patients. In this latter study, *ETFB*, Electron Transfer Flavoprotein Subunit Beta, was identified and replicated as a cardiotoxicity biomarker. ETFB is a flavoprotein subunit located in the mitochondrial membrane that acts as an electron acceptor during energy production. Hence, the *ETFB* variant detected indicates mitochondrial dysfunction involvement in the anthracycline-induced cardiotoxicity mechanisms (38).

All the previous studies included mainly survivors of European ancestry. Individuals with African ancestry are usually at higher risk of non-ischemic cardiomyopathy compared to Europeans. Part of this high vulnerability to cardiomyopathies lies in genetic factors. Nevertheless, PGx of long-term anthracycline-induced cardiotoxicity has been scarcely studied in populations other than Caucasians. Recently, Sapkota and colleagues examined the possible PGx biomarkers of anthracycline-induced cardiotoxicity among 246 African American childhood cancer survivors and compared the findings with 1645 European-ancestry survivors. Both groups were recruited from the St. Jude Lifetime Cohort (SJLIFE) ≥ five years of childhood cancer survivors. This study pointed out a novel locus at 1p13.2 with a significant association with ejection fraction. The detected allele, rs6689879*C, was associated with 5.43-fold cardiomyopathy risk in African-ancestry survivors and a 1.31 fold risk in European-ancestry survivors. Functional assays revealed that the detected allele modulates *PHTF1* (Putative Homeodomain Transcription Factor 1) expression and alternative splicing. The latter gene is involved in transcription regulation. Interestingly, the defective allele was more common in Europeans than in Africans; however, its effect significantly impacted Africans. Accordingly, rs6689879*C was suggested as a cardiotoxicity risk allele for African survivors. In the same study, another locus at chromosome 15 was found to confer increased cardiotoxicity risk in African-ancestry survivors while being absent in their European-ancestry counterparts (39).

Currently, Dexrazoxane is the only available drug approved to prevent anthracycline-induced cardiac damage in children taking high doxorubicin doses. This drug acts through binding iron before entering the cardiomyocyte, thus preventing the iron-anthracycline complex, preventing the free-radical formation and cardiac damage. No other cardioprotective measures are available for ALL children or any other type of cancer (40).

Liposomal doxorubicin formulations have been proposed as alternatives for the conventional formulations with lower cardiotoxicity profiles (41). Liposomal formulations alter drug distribution with a lower accumulation in the heart tissue. However, these formulations are currently approved for limited cases and after the failure of at least one other treatment. The benefits of liposomal doxorubicin in pediatric cancer survivors and its protection against induced cardiotoxicity is still unstudied (42).

The current survivors’ follow-up recommendations include periodic measurements of left ventricular ejection fraction (LVEF) by echocardiography. Recently, alternative methods, like cardiac magnetic resonance imaging (cMRI), are suggested as early screening tools for late-onset cardiotoxicity. Such special applications of non-invasive follow-up tools warrant further research to determine their benefits in reducing long-term consequences for childhood cancer survivors by early detection that enables early intervention (43).

**Figure 3:**
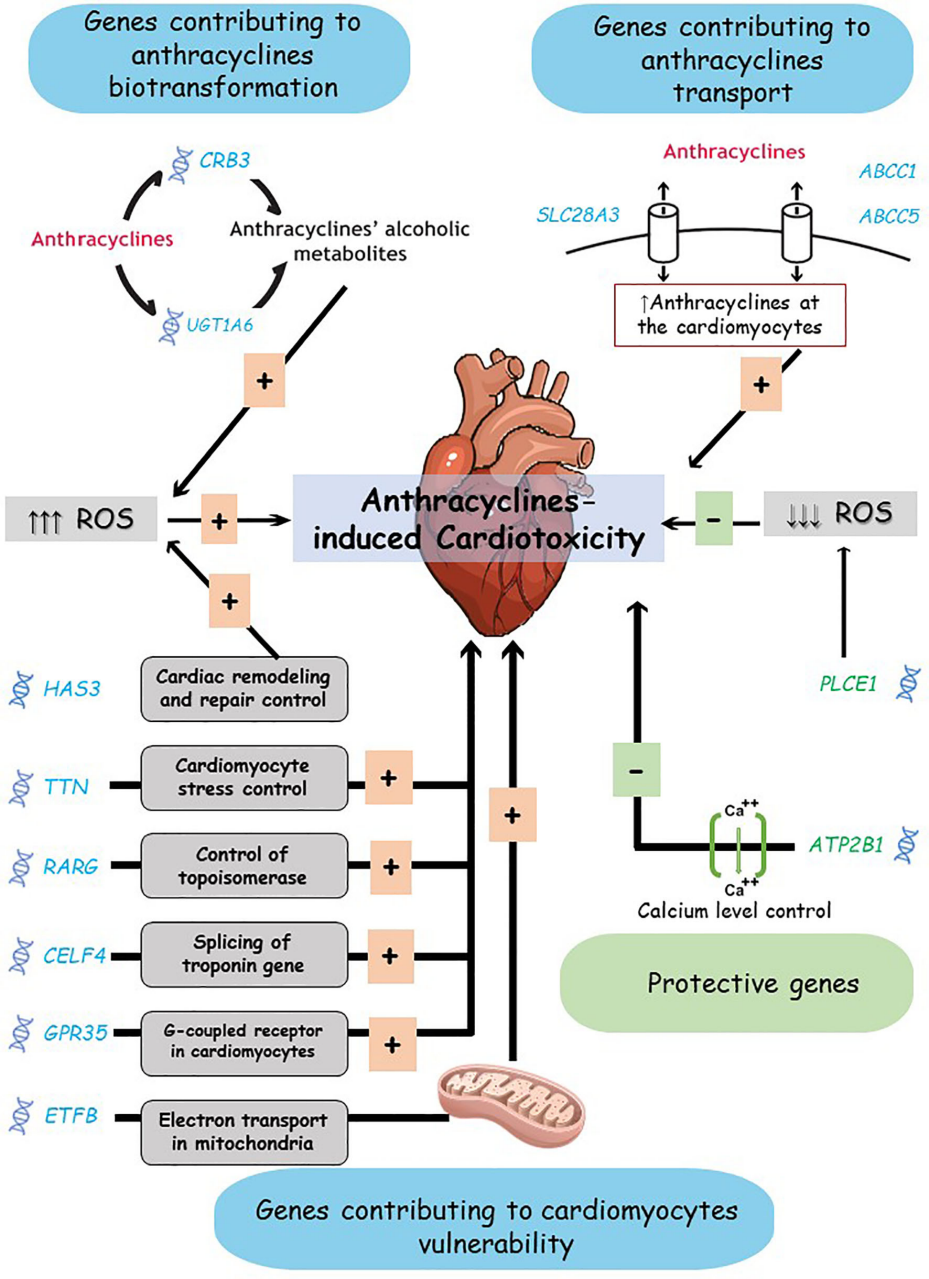
Anthracyclines-induced cardiotoxicity mechanisms and proposed biomarkers. Genes suggested contributing to anthracycline-induced cardiotoxicity risk include: genes active in anthracyclines biotransformation or transport, and genes active in cardiomyocytes vulnerability. The complex interaction between these genes products and the toxicity mechanisms is simplified here. ROS, Reactive Oxygen Species.

To summarize, we listed the most significant findings from studies on chronic anthracycline-induced cardiotoxicity. The detected PGx biomarkers associated with this toxicity lie in two gene groups; genes active in anthracyclines biotransformation or transport and genes that interfere with cardiomyocytes damage and repair; i.e., cardiomyocytes vulnerability to toxicity (**Figure 3**). These findings warrant further confirmatory efforts, which might pave the way for identifying significant biomarkers to be used for individualized therapy.

Notably, more studies have been conducted regarding the acute cardiac events induced during anthracycline treatment, which are not covered here. Nevertheless, biomarkers identified with acute cardiac events are plausible contributors to chronic heart injury. Future work should re-consider all the identified biomarkers and proposed molecular pathways of anthracycline-induced cardiac injury. Such efforts could enable designing more protective agents or tailoring anthracycline doses or surveillance strategies according to the patients’ genome.

### 2.3 Peripheral Sensory or Motor Neuropathy

Chemotherapy-induced neuropathy has complex and multi-factorial mechanisms. Common pathological pathways include oxidative stress, altered calcium homeostasis, membrane remodeling, axon degeneration, and neuroinflammation (44).

Vincristine is the primary neurotoxic agent used in most pediatric ALL protocols. The common adverse event encountered with vincristine use is acute peripheral neuropathy experienced during treatment. However, whether this effect continues to be experienced chronically by the survivors was for a long time uncertain (44). In a study that included 169 cancer survivors, of which 66.9% received vincristine as the sole neurotoxic agent, 53% of the survivors experienced lower limb sensory neuropathy that compromised patients’ quality of life (45). Tay and coworkers reported a vincristine-induced peripheral neuropathy (VIPN) prevalence of 16% among pediatric ALL survivors (46). Other reports indicated a much higher incidence of VIPN that reached up to 78% of children treated with vincristine (47). In one of the most recent analyses considering pediatric ALL survivors, 215 survivors underwent a neurophysiological analysis of motor and sensory nerves. Clinical neuropathy symptoms were noted in 51.6% of patients during the follow-up, with demyelination as the most frequent abnormality. Follow-up was carried at a range of 0.3 to 20.9 years following treatment completion (48). Accordingly, there is increasing evidence supporting the persistence of chemotherapy-neurotoxic consequences in pediatric ALL survivors in the long term.

Several VIPN risk factors were inferred for adult cancer patients, including smoking, diabetes, and other preexisting neuropathy diseases, hypertension, age, and sex. Genetic factors were also proposed with conflicting evidence (49). Many of these risk factors do not exist for children, like smoking, or are rare, like preexisting neuropathy. Hence, genetic factors may play a more significant role for this age group.

Multiple genetic variants have been proposed as probable effectors in developing VIPN from studies applied on mixed cohorts (i.e., adult and children with different types of tumors). The candidate genes include vincristine metabolizing enzymes genes from the cytochrome P450 family (*CYP3A4, CYP3A5, CYP3A7, CYP2C8, CYP2C9*), transporters genes from the ATP binding cassette family (*ABCB1, ABCC1, ABCC2, ABCC4, ABCC5, ABCC3, ABCC10*), and other genes involved in vincristine pharmacodynamics like Centrosomal Protein 72 (*CEP72*). In a recent comprehensive review of the different genetic factors affecting VIPN development, Pozzi and coworkers concluded that the resulting associations are still contrasting, and more focused studies are needed (49).

Focusing on PGx studies carried on pediatric ALL cohorts can highlight some plausible targets for future studies. Children with active CYP3A5 have a 5-fold vincristine clearance compared to low CYP3A5 activity carriers. The same children with the active enzyme exhibited a lower incidence of VIPN (50). Indeed, the same finding was replicated in some studies (51, 52) and failed to replicate in others (53, 54).

*CEP72* is another proposed gene, given that it encodes a centrosomal protein essential in microtubule formation. The first report of the association between *CEP72* and VIPN originated from a GWAS that included 321 pediatric ALL patients (55). The same association was further confirmed by Wright and colleagues in their study on pediatric ALL patients, including 167 cases of grade ≥ 2 neuropathy and 57 controls (54). Together, both studies identified *CEP72* as a possible VIPN biomarker that can be used for individualizing vincristine doses (49). Unfortunately, other studies on similar cohorts failed to replicate this association (56, 57). Nevertheless, in this case, the contradiction could be a result of using different neurotoxicity assessment tools and scores (57). Accordingly, the strength of evidence about the *CEP72* effect on VIPN development warrants further evaluation efforts.

With the lack of consensus on PGx biomarkers that can predict VIPN, other approaches to avoid this drastic adverse event have been suggested. One of these suggested approaches is replacing vincristine with other less neurotoxic agents. Bortezomib, a proteasome inhibitor approved for multiple myeloma, has been suggested as a vincristine alternative for pediatric ALL patients with a reduced neurotoxic profile (58). Joshi and colleagues reported a significant decrease in neuropathy events and a comparable response, measured through relapse rates, for patients switched from vincristine into bortezomib. Nevertheless, this was a small study with some limitations, and further confirmatory randomized controlled trials are needed before approving such approaches (58).

### 2.4 Bone Toxicity

Cancer treatment is known to interfere with bone metabolism. Some of the bone toxicities effects will be occult or subclinical but will be evident with the patient’s aging. These effects put survivors at increased risk of failure to achieve potential peak bone mass and increase their fracture vulnerability in adulthood (59). The prevalence of low bone mineral density (BMD) indicates that osteoporosis occurs in almost 10% of cancer survivors with a median age of 32 years, the exact prevalence of osteoporosis among adult Americans aged 60 to 80 (60). Progression into osteonecrosis can be symptomatic, characterized by severe pain, joint damage, and articular collapse, or remain asymptomatic (61).

Several studies reported an increased risk of bone toxicity for pediatric ALL survivors compared to other cancer types. It was hypothesized that leukemia infiltration directly affects vitamin D metabolism, which might be the causative agent of increased bone toxicity in ALL. However, ALL patients are treated by high doses of corticosteroids and methotrexate, and both agents interfere with bone metabolism (59). Osteonecrosis in ALL usually affects the femoral head, humeral head, shoulder, knee, and ankle (62).

Different groups inferred variable prevalence of long-term low BMD among ALL survivors, ranging between 20% and 50% (63). Contrary to numerous reports, Gurney and colleagues found that low BMD is uncommon in ALL survivors and that the BMD Z-scores, representing the difference in bone density between the study subject and age and sex-matched controls, tend to improve when patients attain young adulthood. The authors concluded that low BMD concerns in ALL survivors are unwarranted (64). However, in this latter study, which included data from SJLIFE, patients were mainly young (median age at ALL diagnosis was five years). Moreover, the BMD change was not adjusted for potential cofounders like vitamin D intake or physical exercise. Accordingly, the findings might not be generalizable, and such conclusions would be better inferred from longitudinal studies measuring BMD over time with fixed intervals (65).

On the contrary, data from the CCSS shows that pediatric HR-ALL and BMT/R-ALL patients treated in the 90s had an increased incidence of a major-joint replacement after 20 years since diagnosis, compared to the 70s ALL survivors (2.7% and 1.8%, respectively *versus* 0.1% in 70s survivors) (2). Accordingly, the effect of ALL-chemotherapy alone on bone is still a vital target for evaluation. Moreover, older studies pointed that osteonecrosis affects up to one-third of ALL survivors, and risk factors include female sex, ALL incidence at age more than ten years, and white race (66). However, fracture risk and BMD deficits are different among patients with similar treatment histories. Accordingly, a genetic contribution in bone toxicity risk was suggested.

#### 2.4.1 Corticosteroids-Induced Bone Toxicity

Since the 1970s, the cumulative corticosteroid dose used during pediatric ALL treatment has increased by 60% to 80%. Dexamethasone replaced prednisone in many protocols because of its longer duration of action, better CNS penetration, and superior protection from central nervous system relapse. However, the former is associated with a higher incidence of side effects (67).

Avascular bone necrosis is a common corticosteroids complication. Fractures are reported in 64% of pediatric ALL patients, which is the highest rate in comparison to other childhood cancer types (68).

Corticosteroids induce changes in the number and function of osteoclasts/osteoblasts enriched with glucocorticoid receptors (GRs). The ligand-GR complexes translocate into the nucleus, initiating up-regulation of pro-apoptotic genes transcription, leading to bone resorption and bone loss (69). Another corticosteroid-induced osteonecrosis mechanism includes creating a hypercoagulable state and microthrombi development at the bone tissue (62). Thus corticosteroids at physiological concentrations play a significant role in bone health, while in higher doses and prolonged use, they will induce unfavorable bone effects (69).

Moreover, hyperlipidemia was found to increase the risk of osteoporosis with a poorly understood mechanism. It was proposed that hyperlipidemia might induce this effect by increasing blood viscosity and reducing bone blood flow (70). Accordingly, corticosteroids lipids alteration is another suggested mechanism of their induced osteoporosis. Furthermore, using asparaginase with dexamethasone probably increases osteoporosis risk through a pharmacokinetic interaction between both drugs and its consequent effect on lipid levels. Nevertheless, this association is still uncertain (70, 71).

In one of the oldest PGx studies exploring corticosteroid-induced osteonecrosis, 12 candidate polymorphisms known for their involvement in osteoporosis were examined. A variant in plasminogen activator inhibitor-1 gene (*PAI-1*), rs6092, showed a positive association with osteonecrosis after adjustment for sex, age, and treatment arm (66). The protein encoded by *PAI-1* is the primary fibrinolytic system’s regulatory controller involved in bone toxicity without a clear understanding of its underlying mechanism (72). Corticosteroids induce an increased production of PAI-1 from bone marrow adipocytes. Accordingly, corticosteroid-induced PAI-1 production is another suggested corticosteroid-induced osteonecrosis mechanism (73). Carrying a genetic variant in *PAI-1* affecting PAI-1 serum levels is expected to increase osteonecrosis risk further (58). Nevertheless, data supporting this association is scarce and limited to short-term follow-up studies. The same biomarker can be considered as a plausible candidate for longitudinal studies in survivors.

In another early PGx study of corticosteroid-induced osteonecrosis, 364 pediatric ALL patients were enrolled. The research aim was to investigate genetic and non-genetic risk factors with a prospective screening of patients for an accurate assignment to case/control groups. The authors reported polymorphisms at *ACP1* (Acid Phosphatase 1) as risk alleles for corticosteroid-induced osteonecrosis. *ACP1* is associated with serum cholesterol levels, and it regulates osteoblasts differentiation. Non-genetic factors concluded from the same study were patients’ age of more than ten years, higher cholesterol levels, lower albumin levels, and higher dexamethasone exposure. These findings underscored the lipid homeostasis alteration as a relevant corticosteroid-induced osteonecrosis mechanism (74).

Interestingly, the same association with other polymorphisms in the same gene, *ACP1*, was found in a recent study that analyzed top hits from preceding GWASs findings. The latter study was different from the older one in the included pediatric ALL population, as the more recent one recruited patients of mixed ancestry. The two studies also differed in corticosteroids type and dose. Moreover, the more recent study supported its findings with a comprehensive explanation of the *ACP1* role as a signaling molecule in bone formation. Accordingly, the *ACP1* gene can be considered a robust candidate biomarker for corticosteroid-induced osteonecrosis in pediatric ALL patients (61).

One of the largest GWASs investigating PGx biomarkers of corticosteroids-induced osteonecrosis included 2285 HR-ALL children as a discovery cohort, 361 children as a validation cohort, and a third independent group of 309 children and adults treated with corticosteroids as a second validation group. In this study, Karol and colleagues pointed out a variant near the glutamate receptor gene, *GRIN3A* (Glutamate Ionotropic Receptor NMDA Type Subunit 3A), as the most significant biomarker. The glutamate receptor variation may contribute to proximal vascular events that interrupt bone’s vascular supply and increase the risk of osteonecrosis in patients treated with corticosteroids (75).

Similarly, but for SR-ALL patients, an independent analysis was carried to investigate corticosteroid-induced osteonecrosis biomarkers. All enrolled patients were under ten years of age and considered at lower risk of developing osteonecrosis. In the discovery subset, 82 developed grade 2-4 osteonecrosis counted as the cases and 287 as controls. A replication set from the same age range was also analyzed. The genome-wide association revealed novel risk variants near *BMP7* (Bone Morphogenetic Protein 7) and *PROX1*-*AS1* (PROX1 Antisense RNA 1). The former gene encodes a protein produced in response to bone damage and affects local bone vasculature. In comparison, *PROX1-AS1* is an RNA gene that alters lipid trafficking through the bone. In the same study, pathway analysis pointed to the glutamate receptor signaling pathway as the top pathway represented by the highest density of genetic variants in the studied cohorts. This latter finding was consistent with preceding results from the HR-ALL group, which determined the same pathway as the top contributing pathway in corticosteroid-induced osteonecrosis. Nevertheless, the glutamate receptor signaling pathway showed a more robust association with the older age group treated with HR-ALL protocols. In contrast, the *BMP7* allele showed a greater osteonecrosis risk in younger ALL patients (76).

Simultaneously, a third GWAS investigated osteonecrosis PGx biomarkers in Asthma children treated with corticosteroids and validated the resulting associations in a pediatric ALL cohort. The selected endpoint for both groups was the BMD Z-score. The highest association retrieved was with rs6461639, an intronic variant located in *RAPGEF5* (Rap guanine nucleotide exchange factor 5) with an unidentified effect (77).

#### 2.4.2 Bone Toxicity Induced by Other Chemotherapy Agents

Methotrexate impairs osteoblasts’ function, number, and responsiveness. After administering a high methotrexate dose, its effect on osteoblast/osteoclast balance may result in bone pain and fractures. However, these effects are reversible, and bone density will improve when the drug is discontinued (78).

Similarly, asparaginase, anthracyclines, and vincristine can affect bone health as an extension of their cytotoxic effects. However, their bone toxicity effects are usually attributed to the synergism with corticosteroids effects (78).

To conclude, chemotherapy affects the pediatric ALL survivors’ bone health in the long term. The manifestations of bone toxicity are not limited to the acute impact on bone density but may affect lifelong bone health. Reduced BMD can be managed by lifestyle changes and attention to other interacting factors like growth hormone deficit and molecular risk factors. The current recommendations for bone health in CCSs include baseline assessment after two years of chemotherapy ends. Other recommendations include adequate calcium and vitamin D supplementation, suitable physical activity, antiresorptive treatment in cases where low BMD is evident, and correction of endocrine alterations if needed (79). However, identifying genetic risk factors can intensify preventive measures in vulnerable patients.

**Figure 4:**
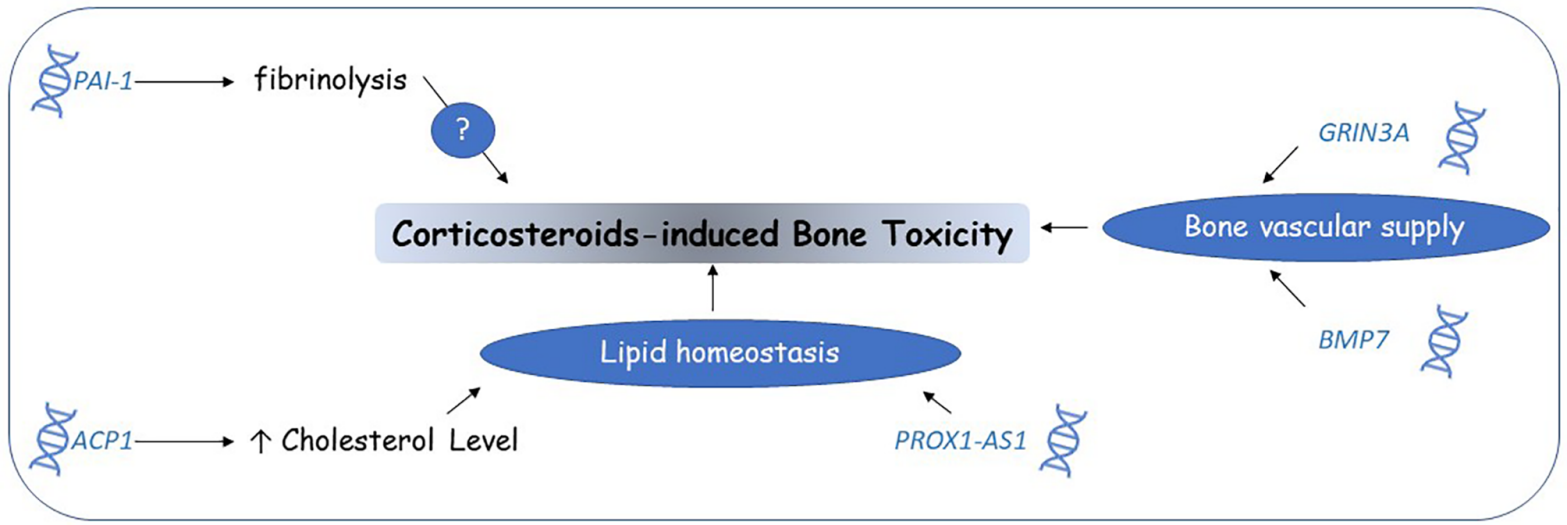
Corticosteroids-induced bone toxicity candidate genomic biomarkers. Pharmacogenomic studies suggested that genetic variants affecting lipid homeostasis and vascular supply to bones might partially explain the mechanisms of corticosteroid-induced bone toxicity. Other mechanisms are still unknown.

Studies on corticosteroid-induced toxicity PGx biomarkers identified multiple candidate genes and pathways (**Figure 4**). Future efforts should focus on validating these findings and confirming the involved mechanisms. *ACP1* and the lipid homeostasis pathway could be the most potential candidates for closer examination.

### 2.5 Hepatic Dysfunction

The evidence of long-term liver damage following chemotherapy in children has been inconclusive, compared to the multiple reports of acute liver injury that resolves at the end of treatment. However, one recent review of the studies conducted on long-term liver injury in CCSs concluded that 1% to 53% of patients show any degree of liver injury years after the treatment end, manifested as an increase in liver enzymes. In the same review, radiotherapy and thioguanine exposure were identified as the treatment-related risk factors encountered by pediatric ALL patients (80).

Pegylated asparaginase has progressively improved the survival rate in children with ALL. It has multiple adverse effects, including hypersensitivity, that warrants replacing it with Erwinia asparaginase. However, abnormal liver function is reported with all asparaginase formulations, causing remarkable elevation of liver transaminase, alkaline phosphate enzymes, and bilirubin (81, 82). Moreover, a similar risk is reported with 6-mercaptopurine and methotrexate, which are indispensable components of ALL protocols. However, all these changes in liver enzymes are usually transient and reversed after therapy discontinuation (83). Significantly, transfusion-associated iron overload, hepatic dysfunction associated with graft-*versus*-host disease, and veno-occlusive disease can contribute to irreversible liver injury (84).

For 6-mercaptopurine (6-MP) and thioguanine, the therapeutic and toxic response is influenced by the thiopurine methyltransferase (TPMT) enzyme encoded by the *TPMT* gene. Any alteration of its activity could cause hepatotoxic metabolites’ accumulation (85). Indeed, there is a strong recommendation for *TPMT* and *NUDT15* genetic testing before starting treatment with thiopurines in ALL children. Dose adjustment recommendations are also available according to genotypes to avoid acute toxicity (86, 87). However, the effect of impaired thiopurine metabolism impairment and personalized treatment on long-term adverse events is not apparent (86).

The major 6-MP metabolite believed to induce gastrointestinal toxicity, including hepatotoxicity, is 6-methylmercaptopurine (6-MMP). Allopurinol acts by altering the metabolism pathways of 6-MP towards less methylation into 6-MMP. Accordingly, allopurinol has been used concomitantly with 6-MP in adults with inflammatory bowel disease refractory to 6-MP. The same approach was suggested as a measure for pediatric ALL patients experiencing acute 6-MP hepatotoxicity. However, such an approach’s long-term impact is still not validated (88).

On the other hand, most studies about methotrexate-induced hepatic injury in cancer children reported its mild nature, transient effect, and low incidence of progression to end-stage liver disease in the absence of co-morbidities. However, leukemia survivors with viral hepatitis who receive high-dose methotrexate are at risk of progressive hepatic dysfunction in adulthood (84).

Multiple PGx biomarkers of methotrexate toxicities were proposed in studies on pediatric ALL patients. Nevertheless, the inconsistency between these studies’ findings excluded inferring a risk allele with a strong evidence association. According to the pharmacogenomics knowledgebase (PharmGKB), *MTHFR* (Methylenetetrahydrofolate Reductase)*, GSTP1* (Glutathione S-Transferase Pi 1), and *ABCB1* are the proposed genes for methotrexate toxicities but with limited supporting evidence (89).

There is growing evidence of omega-3 fatty acids’ effects in improving non-alcoholic fatty liver disease, which suggested using these agents to mitigate methotrexate-induced liver injury. In a clinical trial that included 70 pediatric ALL patients, omega-3 fatty acid at a 1000 mg/day dose was well-tolerated and correlated with better liver function parameters compared to placebo. However, such approaches need validation in more extensive trials. The long-term benefit of such protective measures should be evaluated as well (90).

### 2.6 Visual Changes

Cancer survivors report neurosensory complications impacting ocular, auditory, speech, and olfactory systems. Whelan and colleagues described the increased risk of eye-related disorders in a report from 8,507 children from the CCSS data with a follow-up spanning 25 years. The studied defects included cataracts, glaucoma, blindness, double vision, and dry eye. In the same report, the identified risk factors were radiation and corticosteroids exposure (91).

Few studies have evaluated the visual deterioration or other treatment-induced eye defects, specifically in pediatric ALL survivors. One recent report from 59 ALL survivors compared multiple ocular parameters, including visual acuity, accommodation amplitude, and other parameters, with 48 age, sex, and race-matched controls. The survivors demonstrated significant differences, with substantial premature ocular aging features among survivors (92).

Apart from this limited data from pediatric ALL survivors, no more reports about ocular health are available. More research on ALL-treatment long-term effects on the ocular system is needed.

### 2.7 Body Mass Index Changes, Obesity, and Metabolic Syndrome

Obesity, defined by a body mass index (BMI) ≥ 30 kg/m^2^, is a common long-term consequence of pediatric ALL treatment. The CCSS data shows an increase of 20% and 50% in obesity prevalence among male and female ALL-survivors, respectively, compared to the general population (93). A more recent meta-analysis, reporting on 9223 ALL survivors from 47 studies, indicated a significantly higher BMI than the reference population. Prevalence ranged between 29% and 69% in 11 studies where patients were off treatment between 5 and 9 years. In contrast, for studies including survivors off-treatment for at least ten years, obesity prevalence ranged between 34% and 64% (94).

Accordingly, obesity is a significant sequela that affects survivors’ life in the long term. Given its association with the risk of multiple morbidities, including hypertension, type 2 diabetes, and cardiovascular diseases, weight gain induced by ALL-treatment has been repeatedly investigated (95). The contribution of obesity in cardiovascular morbidities and mortalities is of additional concern, considering that the same patients have received potential cardiotoxic chemotherapy (96).

Weight gain risk factors among ALL-survivors were explored by multiple groups with conflicting outcomes. A study that included 1,638 patients treated with the CCG-1961 protocol, found significant risk factors include female gender, age, race, baseline BMI, and glucose toxicity during induction. In contrast, cranial radiation was not an obesity risk factor in the same trial (97). The latter finding was contrary to multiple other groups that designated CRT and radiation doses as obesity risk factors in ALL-survivors (98, 99). Similarly, the effect of gender on weight gain risk was inconsistent. Some studies reported a higher prevalence among females (97, 99, 100) compared to other groups that found no difference between genders. Due to the multiple inconsistencies between the predisposing risk factors in different cohorts, the most substantial evidence can be based on a meta-analysis. In their meta-analysis of 47 extensive studies, Zhang and colleagues reported that obesity prevalence among ALL-survivors was independent of gender, age at diagnosis, or CRT receipt (94). On the other hand, it is still unclear if dexamethasone can cause a more significant increase in weight gain than prednisone and whether the effect of cortisone is only transient or long-lasting (96).

Indeed, effective investigation and intervention planning require understanding the etiology of obesity and identifying vulnerable patients to enable follow-up and protective measures. During treatment, low physical activity due to hospitalization, diminished exercise capacity because of cancer-induced or treatment-induced pain (e.g., vincristine-induced neuropathy), and increased energy intake, are all lifestyle factors contributing to weight gain (96). However, the same elements cannot justify the prevalence of obesity among survivors after decades of diagnosis and treatment cessation.

Molecular studies pointed out that CRT and chemotherapy can induce obesity in survivors with distinct mechanisms (95). Radiotherapy can provoke hypothalamic damage, which might dysregulate food intake control or cause hormonal deficiency. Both are suggested mechanisms for long-term weight gain for ALL-survivors treated with CRT. Nevertheless, conflicting evidence supports the association between CRT or the radiation dose and survivor’s obesity and the mechanism behind this association (96).

The continued observation of long-term obesity among not irradiated survivors suggested that chemotherapy is an independent risk factor. Currently, the proposed mechanisms include the central effects of chemotherapeutic agents leading to growth hormone deficiency, which predisposes survivors to metabolic consequences, including weight gain. Moreover, corticosteroids impact appetite and energy uptake/expenditure (96). Furthermore, corticosteroid exposure during early childhood may induce epigenetic changes, which predispose to long-term body composition changes (101).

The complexity of the BMI trait and the hundreds of identified loci which explain about 6% only of its variability, may justify the low available data in this topic. Indeed, a limited number of studies explored genomic biomarkers of obesity in pediatric ALL survivors. In a study of 1996 cancer survivors followed up to median age of 32.4 years; 47% and 29.4% of patients treated with or without CRT, respectively, were obese. The results confirmed the increased odds of obesity in adults treated with corticosteroids during childhood. In the same study, the whole-genome analysis yielded 166 SNPs associated with a BMI ≥ 30 kg/m^2^. The strongest associations were found in neuronal growth, repair, and connectivity genes, namely *NALF1* (NALCN Channel Auxiliary Factor 1 genes, previously known as *FAM155A)*, *SOX11* (SRY-Box Transcription Factor 11), *CDH18* (Cadherin 18), and *GLRA3* (Glycine Receptor Alpha 3) (102).

One recent trial investigating 1458 adult survivors of pediatric ALL mostly treated with CRT identified two novel loci; *LINC00856* (Long Intergenic Non-Protein Coding RNA 856):rs575792008 and *EMR1* (EGF-Like Module Receptor 1):rs62123082. A later comparison of the detected associations and loci known to affect BMI variance in the general population concluded similarities between the heritability factors of obesity in ALL survivors and the general population. Nevertheless, CRT modified the effect of these variants on the survivors’ BMI. The low number of patients treated with chemotherapy alone precluded finding significant associations in the same study, although high BMI was still reported in this subgroup (103).

Interestingly, the N363S polymorphism in the glucocorticoid receptor gene, *NR3C1*, known to increase corticosteroid toxicity vulnerability, was investigated in a group of pediatric ALL survivors. The results indicated the increased exposure of the N363S carriers to several corticosteroid long-term toxicities. The same study did not include obesity indicators as one of its collected data; nevertheless, the same polymorphism has been identified as a risk allele for corticosteroid-induced obesity in preceding studies on non-cancer patients. Herein, the same allele warrants further investigation in pediatric-ALL survivors, which might enable a better corticosteroid individualized therapy (104).

Emerging evidence suggests that epigenetic changes correlate better than genomic variants to BMI and other obesity quantitative traits. DNA methylation is an epigenomic event that involves the reversible and heritable attachment of a methyl group to a nucleotide, occurring mainly at the CpG islands in the gene promotors. DNA methylation is affected by genetic and environmental factors, which integrate into a phenotype impact. Vise-versa, the epigenetic changes, like DNA-methylation, will affect gene expression (105). In the pathogenesis of a complex trait like obesity, DNA methylation can explain the phenotype’s cause and consequence. Few epigenome-wide studies (EWAS) have been conducted to explore the molecular mechanisms of obesity on samples from the general population. These studies concluded several DNA-methylation hotspots which correlate to obesity (105, 106). Using a similar approach, Lupo and colleagues performed an EWAS on pediatric ALL survivors, with BMI as the phenotype. The study, which included 96 adult survivors of pediatric ALL, concluded that patients not treated with CRT share a DNA-methylation profile similar to the DNA-methylation profile of obese from the general population but distinct from those treated with CRT. These findings support the growing body of evidence that obesity mechanisms in CRT exposed and un-exposed (chemotherapy-alone) patients are different; nevertheless, they are similar to general population obesity mechanisms (95).

Importantly, central obesity is considered one of the metabolic syndrome disorders (MS). Other components of MS include elevated plasma glucose, insulin resistance, hypercholesteremia, hypertension, and prothrombotic/proinflammatory state (96). Childhood ALL survivors treated with chemotherapy alone are at increased risk of MS by almost two folds compared to their age-matched controls. In general, regardless of the treatment they received, ALL survivors are at an increased risk of MS, which warrants closer follow-up for these individuals (107).

Moreover, diabetes was among the treatment complications with high frequency in the data collected from the CCSS group with a frequency of 4% and 5.7% for HR-ALL and BMT/R-ALL, respectively (Dixon 2020). Diabetes can result from asparaginase toxicity. Asparaginase can induce pancreatitis, which can progress into a chronic form leading to insulin-dependent diabetes mellitus and exocrine function impairment (71). Endocrine late effects frequently occur in the long term after the end of cancer treatment. The cumulative risk of developing an endocrine disorder increases steadily over time in childhood cancer survivors. The current COG-LTFU guidelines recommend screening patients treated with high-risk therapies (i.e., high dose radiation and alkylating agents). Nevertheless, recent evidence supports the increased incidence of endocrinopathies in childhood cancer survivors treated with non-high-risk therapies (108).

To conclude, obesity forms a unique modifiable factor. Data about its molecular mechanisms and the genetic contribution towards obesity and MS progress is scarce. Given the complexity of identifying molecular biomarkers for patients at risk, epigenetic studies can reveal more information about molecular mechanisms of obesity in cancer survivors and form a hot spot for future research.

### 2.8 Infertility and Sex Hormone Deficiency

Substantial evidence supports the impact of gonadal or cranial radiotherapy on gonads and fertility of cancer survivors. Moreover, the gonads are vulnerable to cytotoxic effects of anticancer therapy. With the gradual reduction in radiotherapy and replacing it with higher intensity chemotherapeutics, the long-term effects of these newer protocols on survivors’ fertility and sexual health are understudied (101, 102).

Male gonads are usually more prone to cancer therapy effects than females. Oligospermia and harmful changes in the sperms’ quality are commonly reported, matched to fewer reports on induced ovarian insufficiency, and oocyte number depletion in females (109).

In a report from more than 10,000 survivors of the CCSS dataset who were not exposed to radiotherapy, male survivors were less likely to sire a pregnancy than siblings. Similarly, females were less likely to report pregnancy or live birth when compared to siblings. Females who did not report pregnancy before the age of 30 were less likely to have any pregnancy by 45. This cohort’s treatment risk factors included alkylating agents’ cumulative doses, cisplatin treatment for males, and busulfan treatment in females. Nevertheless, this data describes the survivors’ pregnancy incidence rather than directly assessing gonadal function. Accordingly, a percentage of the results could be reflecting personal choices, or psychosocial factors, rather than biological functions (110).

Cyclophosphamide, used in some pediatric ALL protocols, has a gonadal toxic effect on high doses. Moreover, harmful gonadal effects are reported with cytarabine and doxorubicin, but to less extent. However, ALL patients usually suffer less gonadal toxicity than other types of tumors (109). To determine the effect of different ALL treatment modalities on gonadal function, Krawczuck-Rybak and colleagues analyzed multiple parameters in a group of pediatric ALL survivors of both sexes. The analysis encompassed measuring follicle-stimulating hormone (FSH), luteinizing hormone (LH), and inhibin B, with testosterone for males and estradiol with anti-Mullerian hormone (AMH) for females. Despite the low gonadal toxicity attributed to the administered agents, the findings indicated significant hormonal changes in ALL survivors. For males, elevated levels of FSH and LH and decreased inhibin B levels, as indicators of lower spermatogenesis, characterized the survivors, with more predominant changes in the HR-ALL group. Only lower AMH levels in the HR-ALL group were reported in females, reflecting lower ovarian reserve (109).

Cryopreserved ovarian/testicular tissue before cancer chemo/radiotherapy has been suggested as a prophylaxis measure. Multiple researchers investigated the possibility and safety of implanting these tissue after the recovery of ALL patients. Unfortunately, re-implanting spermatogonia stem cells (SSC) or oogonial stem cells (OSC) was associated with a potential risk of disease recurrence due to the infiltration of leukemic cells into the ovarian/testicular tissues (111, 112). There is plenty of research on *in vitro* auto-transplantation that reinforces stem cell preservation practice. Detecting the round spermatids taken from the testicular biopsy can give another hope for round spermatid injection (ROSI). With the pros and cons of fertility cell preservation and the complexity of handling cancer patients, it is crucial to have a multidisciplinary collaboration to achieve superior overall fertility outcomes for survivors (112).

### 2.9 Neurocognitive Effects

Long term effect of cancer treatment on the patient’s neurocognitive abilities has been usually ascribed to the CRT impact. In pediatric ALL, CNS-directed prophylaxis is indispensable. The high probability of leukemic cell infiltration to the CNS puts patients at an elevated risk of CNS relapse and associated severe morbidity and mortality. Older regimens used a combination of CRT and intrathecal methotrexate as CNS prophylaxis. However, CRT intensity has been reduced, and then it was omitted for most ALL patients after recognizing its detrimental effects on intellectual and learning capabilities (68, 113).

Nevertheless, cognitive function deterioration is encountered with patients not exposed to CRT and is attributed mainly to the CNS prophylactic chemotherapy (113). The suggested mechanisms include chemotherapy-induced suppression of cell proliferation and an increase in neuroinflammation (114). Other proposed mechanisms are loss of phospholipids affecting white matter architecture and the disruption of the developing neural networks in children (115).

The measurement of neurocognitive changes is usually determined through educational progress and long-term job achievements. In a study that included 129 childhood cancer survivors treated by the 1990s treatment protocols, additional school resources were needed by 30% of the survivors, from which 50% were pediatric ALL survivors mostly treated with chemotherapy alone. In the same study, 10% of participants required attention deficit hyperactivity disorder (ADHD) prescription medication, double the national average of children between 2-17 years of age (68). Other studies reported an increased cumulative dose of intravenous methotrexate (doses more than 4.3mg/m2) is associated with an increased risk of inattention-hyperactivity (116).

A recent analysis of the CCSS dataset analyzed 1207 survivors of pediatric ALL who were treated with chemotherapy alone and were all at least 18 years old at the follow-up assessment. The participants and their non-cancer siblings completed neurocognitive surveys. The results revealed an increased neurocognitive, efficiency, and memory impairment in the survivors compared to their same-sex siblings. Methotrexate was associated with neurocognitive impairment in both sexes, while dexamethasone was associated with memory impairment only in males. The difference in dexamethasone effect among sexes might result from an interaction between sex hormones and external steroids. This study concluded that neurocognitive impairment risk factors are sex, methotrexate and/or dexamethasone exposure, and chronic conditions, like pulmonary diseases (115).

These recent results were preceded by several other reports and reviews that shed light on ALL treatment’s critical effects on the neurocognitive functions during adulthood (113). However, this research body did not elucidate the underlying mechanisms or suggested any pharmacological targets or interventional approaches. More research needs to be performed in this area to understand the mechanisms and design effective interventions.

## 3 Conclusions and Future Directions in Long-Term Sequela of Pediatric ALL Treatment

As illustrated in the above sections, several long-term effects have been attributed to pediatric ALL therapy. Despite the lower incidence of life-long consequences in newer protocols, clinicians should keep these events in mind during the early treatment phases.

The reported long-term effects can be categorized into:

1. Rare events with devastating consequences, like SMNs.
2. Common adverse events with severe late manifestations, like cardiotoxicity.
3. Common events that can be modulated by lifestyle changes, like obesity and bone density impairment.
4. Adverse events that are not well studied in the long term, like VIPN, ocular effects, fertility impairment, neurocognitive impairment, and liver function impairment.

Accordingly, each of the previous groups requires a different approach. **Table 1** summarizes the recommendations we concluded from the reviewed literature.

**Table 1:** The revised long-term adverse events of pediatric ALL treatment and the recommended approaches to respond to the latest findings.

	Long-term adverse event	Latest findings	Recommended approach
Rare and severe	Secondary malignant neoplasms (SMNs)	Patients carrying *TP53* are at an increased risk of SMNs.	To initiate early detection of *TP53* mutations in the routine tests at diagnosis. To add survivors with *TP53* mutations to the high-risk group of patients that need closer surveillance. Using less-genotoxic agents for these patients should be evaluated.
		*POT1* variants are possible biomarkers of secondary thyroid tumors.	Confirmatory studies may lead to adding *POT1* mutation carriers to the high-risk group of survivors.
		Anthracyclines increase the risk of breast cancer in survivors.	To apply research to confirm this association and elucidate its mechanism followed by preventive strategies (e.g., early detection, treatment individualization).
Common and severe	Anthracycline induced cardiotoxicity (AICT)	Several candidate genes are proposed biomarkers of AICT like *CRB3, SLC28A3, UGT1A6, ABCC1*, and others.	Research is needed to confirm the suggested associations and establish a list of biomarkers that can be tested early after diagnosis. Survivors with confirmed cardiotoxicity genetic vulnerability require closer follow-up.
		Africans harbor distinct AICT biomarkers.	Future research should consider different ethnicities.
		Currently, dexrazoxane is the only available drug to prevent cardiotoxicity when high doses of anthracyclines are used.	Other protective agents can be developed to target the variable AICT pathogenesis mechanisms.
		MRI can identify cardiac damage earlier than the conventionally used ones	Confirmatory research should be applied to children to measure the benefits of this approach in the long term.
		Liposomal anthracyclines induce fewer cardiotoxic effects in adults	Research is needed to approve the safety of these formulations for children.
Common and can be modulated by lifestyle changes	Bone toxicity	Some genetic variants are identified as possible biomarkers of corticosteroid-induced bone toxicity.	Confirmatory studies are required to confirm the suggested associations. Early genetic testing for the confirmed biomarkers can identify patients who might need lower corticosteroid doses or benefit from replacing dexamethasone with prednisolone. To recommend lifestyle changes for patients with confirmed risk variants (e.g., Vitamin D supplements, physical exercises).
	Obesity and metabolic syndrome	Some genetic variants are identified as possible biomarkers of obesity vulnerability.	Confirmatory studies are required to confirm the suggested associations. Early genetic testing for the confirmed biomarkers can identify patients who might need lower corticosteroid doses or benefit from replacing dexamethasone with prednisolone. To recommend lifestyle changes for patients with confirmed risk variants (e.g., adjusted diets, physical exercises).
		Chemotherapy-induced obesity is probably associated with genetic biomarkers similar to those reported from common obesity in contrast to radiation-induced obesity	More research is needed for a better understanding of chemotherapy-induced obesity mechanisms to enable designing better protective measures.
Rarely studied events	Liver toxicity	Few reports of long-term toxicity	More investigation is needed on survivors to determine the prevalence of these events, understand their pathophysiology, and design protective and follow-up measures.
	Ocular effects	Very few reports	
	Neurocognitive effects	Very few reports	

Undoubtedly, achieving a cure is the caregivers’ priority at the acute stage; nevertheless, the following suggested approaches have the potential to present life-long improved outcomes:

1. Genetic testing to predict vulnerable patients: Testing for genetic predisposition mutations, like *TP53* indicating susceptibility to secondary cancers, would identify patients with high risk for individualized follow-up measures. Likewise, when more vulnerability biomarkers of other toxicities are confirmed, the proved variants warrant early testing during the first phases of treatment. Inclusion of such results in patients’ electronic files would provide them with the opportunity of individualized follow-up.
2. Delineating the underlying cellular mechanisms of toxicity and long-term effects of those therapies would provide new opportunities for minimizing them.
3. PGx guided drug choice and dose adjustment: When adequate evidence supporting toxicity risk biomarkers is accumulated, prospective studies of genotype-guided dose adjustment will pave the way for personalized dosing to avoid long-term sequelae.
4. Targeted therapies instead of chemotherapies: Theoretically, targeted therapies can spare patients from the off-target effects of traditional cytotoxic chemotherapies. Currently, targeted therapies are under evaluation for relapsed ALL. These agents are still not the standard of care yet; however, they might be soon (117). As soon as targeted therapy strategies are adopted, longitudinal studies will be needed to explore their long-term toxicities.
5. Adjusting follow-up recommendations: There is an immense need to update the current pediatric-ALL follow-up recommendations to accommodate the latest findings. Utilizing the newer techniques of massive data analysis and artificial intelligence can facilitate interpreting the results to apply optimized precision follow-up practices.

To conclude, throughout this literature review, we highlighted the novel findings at understanding long-term sequela of pediatric ALL treatment. We shed light on the available data at the molecular level and the knowledge gaps in the field. Nevertheless, it is still early for the majority of the illustrated findings to impact clinical practice.

## Author Contributions

ZA-M: Conceptualization, literature review, writing, and final editing. MA: Literature review and writing. BA: Fund acquisition, conceptualization, and manuscript revising. All the authors contributed to the final manuscript editing.

## Funding

This research was supported by the UAE Ministry of Education funding (grant 21M139).

## Conflict of Interest

The authors declare that the research was conducted in the absence of any commercial or financial relationships that could be construed as a potential conflict of interest.

## Publisher’s Note

All claims expressed in this article are solely those of the authors and do not necessarily represent those of their affiliated organizations, or those of the publisher, the editors and the reviewers. Any product that may be evaluated in this article, or claim that may be made by its manufacturer, is not guaranteed or endorsed by the publisher.
